# Supplementary material for: Deletion of wfs1 Impairs Oligodendrocyte Precursor Cells Dorsal Distribution and Myelination Through the wfs1-hmgcs1 Axis in Zebrafish
Source: Biology (Basel). 2026 Aug 16;15(16):1402. doi: 10.3390/biology15161402 (PMC13510151; doi:10.3390/biology15161402)
Supplement: Supplementary file 1 [file biology-15-01402-s001.zip › File S1-Transcriptome sequencing data and differentially expressed genes (DEGs) in wfs1-- zebrafish at 5 dpf/1.GO/wfs1KOvsWT/GSEA/WT/SITE_OF_DNA_DAMAGE/SITE_OF_DNA_DAMAGE(GO_0090734).html]

Details for gene set SITE\_OF\_DNA\_DAMAGE(GO:0090734)[GSEA]


|  || Dataset | deg.sample |
| Phenotype | sample.cls |
| Upregulated in class | WT |
| GeneSet | SITE\_OF\_DNA\_DAMAGE(GO:0090734) |
| Enrichment Score (ES) | -0.44482464 |
| Normalized Enrichment Score (NES) | -1.725009 |
| Nominal p-value | 0.034816246 |
| FDR q-value | 0.33576512 |
| FWER p-Value | 0.827 |
Table: GSEA Results Summary

  

Fig 1: Enrichment plot: SITE\_OF\_DNA\_DAMAGE(GO:0090734)
  
Profile of the Running ES Score & Positions of GeneSet Members on the Rank Ordered List

  

| PROBE | DESCRIPTION   (from dataset) | GENE SYMBOL | GENE\_TITLE | RANK IN GENE LIST | RANK METRIC SCORE | RUNNING ES | CORE ENRICHMENT || 1 | 557127 | si:dkey-119f1.1 |  |  | 1897 | 1.415 | 0.0275 | No |
| 2 | 406820 | actr2a |  |  | 3314 | 0.969 | 0.0427 | No |
| 3 | 797304 | smc6 |  |  | 3819 | 0.865 | 0.0802 | No |
| 4 | 406637 | mre11a |  |  | 3853 | 0.859 | 0.1320 | No |
| 5 | 553453 | samhd1 |  |  | 5421 | 0.638 | 0.1221 | No |
| 6 | 563175 | smarcad1b |  |  | 7962 | 0.426 | 0.0686 | No |
| 7 | 100329327 | rfwd3 |  |  | 8410 | 0.410 | 0.0798 | No |
| 8 | 100149820 | si:ch211-134m17.9 |  |  | 10313 | 0.284 | 0.0376 | No |
| 9 | 65226 | rpa2 |  |  | 10674 | 0.262 | 0.0425 | No |
| 10 | 393578 | ufl1 |  |  | 11203 | 0.233 | 0.0402 | No |
| 11 | 324358 | si:ch211-200e2.1 |  |  | 11863 | 0.197 | 0.0317 | No |
| 12 | 554861 | actr2b |  |  | 15691 | 0.012 | -0.0876 | No |
| 13 | 393196 | rpa3 |  |  | 16744 | -0.024 | -0.1192 | No |
| 14 | 557043 | mb21d1 |  |  | 17109 | -0.037 | -0.1283 | No |
| 15 | 335495 | parp3 |  |  | 17558 | -0.056 | -0.1389 | No |
| 16 | 393599 | ddb1 |  |  | 20019 | -0.167 | -0.2059 | No |
| 17 | 503536 | rbbp8 |  |  | 20216 | -0.175 | -0.2012 | No |
| 18 | 404050 | rnf8 |  |  | 22378 | -0.288 | -0.2513 | No |
| 19 | 492631 | zgc:193538 |  |  | 23392 | -0.354 | -0.2613 | No |
| 20 | 795748 | rnf169 |  |  | 23757 | -0.377 | -0.2495 | No |
| 21 | 334081 | rnf168 |  |  | 23936 | -0.388 | -0.2312 | No |
| 22 | 100002184 | hpf1 |  |  | 30743 | -1.716 | -0.3392 | Yes |
| 23 | 100037341 | zgc:162895 |  |  | 30823 | -1.781 | -0.2321 | Yes |
| 24 | 327197 | vcp |  |  | 31772 | -4.315 | 0.0037 | Yes |
Table: GSEA details
[plain text format]

  

Fig 2: SITE\_OF\_DNA\_DAMAGE(GO:0090734)
  
Blue-Pink O' Gram in the Space of the Analyzed GeneSet

  

Fig 3: SITE\_OF\_DNA\_DAMAGE(GO:0090734): Random ES distribution
  
Gene set null distribution of ES for
 **SITE\_OF\_DNA\_DAMAGE(GO:0090734)**
